# Approaches to Visualising Endocytosis of LDL-Related Lipoproteins

**DOI:** 10.3390/biom12020158

**Published:** 2022-01-18

**Authors:** Halima Siddiqui, Nikita Yevstigneyev, Golnoush Madani, Sally McCormick

**Affiliations:** 1 Department of Biochemistry, School of Biomedical Sciences, University of Otago, Dunedin 9016, New Zealand; samha141@student.otago.ac.nz (H.S.); yevni454@student.otago.ac.nz (N.Y.); golnoush.madani@otago.ac.nz (G.M.); 2 HeartOtago, School of Biomedical Sciences, University of Otago, Dunedin 9016, New Zealand

**Keywords:** endocytosis, transcytosis, macropinocytosis, low-density lipoprotein, lipoprotein(a), confocal microscopy, electron microscopy, live-cell imaging, TIRF

## Abstract

Endocytosis is the process by which molecules are actively transported into cells. It can take on a variety of forms depending on the cellular machinery involved ranging from specific receptor-mediated endocytosis to the less selective and actin-driven macropinocytosis. The plasma lipoproteins, which deliver lipids and other cargo to cells, have been intensely studied with respect to their endocytic uptake. One of the first molecules to be visualised undergoing endocytosis via a receptor-mediated, clathrin-dependent pathway was low-density lipoprotein (LDL). The LDL molecule has subsequently been shown to be internalised through multiple endocytic pathways. Dissecting the pathways of lipoprotein endocytosis has been crucial to understanding the regulation of plasma lipid levels and how lipids enter cells in the arterial wall to promote atherosclerosis. It has also aided understanding of the dysregulation that occurs in plasma lipid levels when molecules involved in uptake are defective, as is the case in familial hypercholesterolemia (FH). The aim of this review is to outline the many endocytic pathways utilised for lipoprotein uptake. It explores the various experimental approaches that have been applied to visualise lipoprotein endocytosis with an emphasis on LDL and its more complex counterpart, lipoprotein(a) [Lp(a)]. Finally, we look at new developments in lipoprotein visualisation that hold promise for scrutinising endocytic pathways to finer detail in the future.

## 1. Introduction

Endocytosis allows cells to actively internalise molecular cargo, such as proteins, lipoproteins, hormones, nutrients, and pathogens [1,2]. It is the cells’ means of sampling environmental cues to drive cellular actions, such as nutrient uptake, regulation of cell signalling, membrane component turnover and recycling. Several pathways of endocytosis exist depending on the cargo, receptor involvement and cellular machinery utilised. Of the receptor-mediated pathways, clathrin-mediated endocytosis [3] is the most well-characterised constituting the major route for internalisation of many cargos. Non-clathrin-mediated pathways that are driven by other endocytic proteins, such as endophilin or caveolin, have also been documented [4]. The receptor-mediated pathways result in the receptor and its specific cargo on the plasma membrane being internalised within adaptor and coat-protein-rich vesicles. Once internalised, these vesicles engage with various sorting endosomes to determine cargo destination such as lysosomal degradation, recycling or exocytosis. Transcytosis is a form of receptor-mediated endocytosis, which is coupled with exocytosis to transport cargo across the interior of a cell [5]. Less selective forms of endocytosis such as pinocytosis are receptor-independent and constitute a form of non-selective “cellular drinking” in which solutes and nutrients are ingested through the formation of small vesicles [6]. Macropinocytosis is a complex form of pinocytosis in which actin-dependent membrane remodelling driven by cellular signalling allows for the formation of larger vesicles, or macropinosomes, containing fluid and associated cargo [7].

Plasma lipoproteins are lipid and protein complexes made in the intestine and liver to transport lipids from sites of absorption, or synthesis, to peripheral tissues. Their amphipathic nature makes them the ideal transport vehicle for delivering various other payloads, including proteins, vitamins, hormones, microRNAs, drugs and imaging agents [8]. They are also ideal macromolecules to study the various forms of endocytosis. Indeed, low-density lipoprotein (LDL) was one of the first molecules shown to undergo endocytosis and the study of its uptake by the low-density lipoprotein receptor (LDLR) established the classic route of receptor-mediated endocytosis [9]. Since then, the various classes of lipoproteins have collectively been shown to be internalised by almost every known form of endocytosis. In particular, LDL lends its hand to uptake by multiple pathways. Discoveries in the field of lipoprotein endocytosis have been made using a broad range of technologies, from high sensitivity ^125^I binding assays for tracking ligand binding and degradation, to multiple microscopy-based imaging techniques. These include electron microscopy to visualise the fine structural details and high-resolution confocal imaging for tracking multiple proteins and assessment of intracellular location. More recently, live cell imaging has been developed yielding additional spatial and temporal information about lipoprotein endocytosis. Lipoprotein uptake by the various pathways of endocytosis and the approaches to visualise this is the subject of this review.

## 2. Forms of Endocytosis

**Figure 1:**
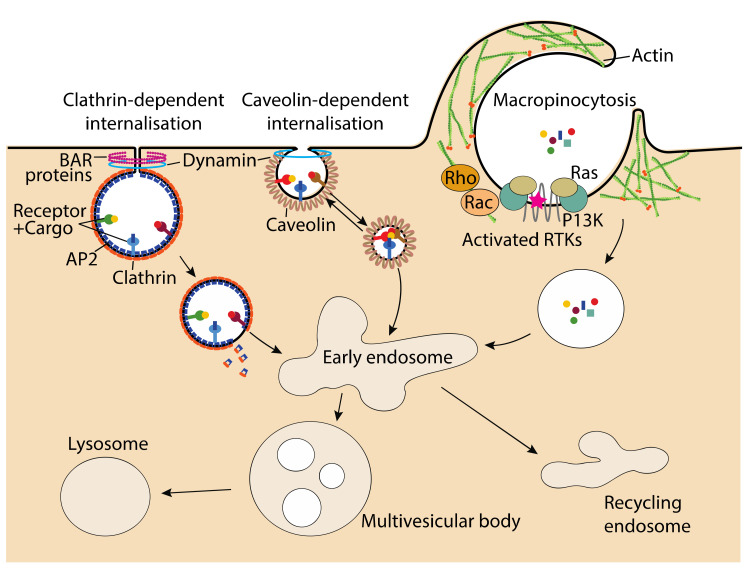
Major routes of endocytosis involved in lipoprotein uptake. Clathrin-dependent endocytosis employs clathrin recruited by adaptor protein, AP2, along with receptors to bind and concentrate cargo in coated pits. Clathrin polymerisation promotes membrane invagination, and along with the BAR proteins, stabilise vesicle formation. Dynamin recruited by the BAR proteins facilitates release of the vesicle. Caveolin-dependent endocytosis utilises the cholesterol-binding caveolin protein to form caveolae (little caves) in which receptors are concentrated. Binding of the cargo to its receptor initiates budding and release of the caveolae via various kinases and phosphatases leading to internalisation. Macropinocytosis constitutes a non-specific form of cargo capture which is driven by calcium sensing or activation of receptor tyrosine kinases (RTKs) to drive actin polymerisation via Rac and Rho GTPases. This promotes membrane ruffling and formation of a macropinosome which imbibes localised cargo and associated fluid. Internalised cargo, regardless of the endocytic route of entry, is then trafficked and sorted via the endosomal system to various destinations. Transcytosis (not shown) is usually initiated by one of these three endocytic routes followed by trafficking of the encapsulated cargo across the cell and exit via exocytosis.

There are many different endocytic processes that are used by cells to internalise their cargoes. The types of endocytoses employed for lipoprotein uptake, clathrin-mediated, caveolae-mediated and macropinocytosis are depicted in Figure 1. Another form of endocytosis utilised for lipoprotein uptake, transcytosis (not shown), is initiated by one of these three forms, followed by the cargo being trafficked across the cell to be exocytosed.

### 2.1. Clathrin-Mediated Endocytosis

Clathrin-mediated endocytosis relies on clathrin, a self-polymerising protein, recruited to the plasma membrane via adaptor proteins such as assembly polypeptide 2 (AP2) [10]. The AP2 protein is recruited to phosphatidylinositol 4,5-bisphosphate (PIP_2_) enriched regions of the plasma membrane where it binds to the cytoplasmic tail of cargo-bound receptors [11]. Along with receptor-specific adapter proteins, AP2 concentrates the cargo and recruits clathrin to the growing AP2 complex to form clathrin-coated pits in the membrane [11]. The recruited clathrin polymerises to stabilise the curvature of the membrane while simultaneous polymerisation of actin fibres promotes membrane bending and invagination [11,12]. The Bin/Amphiphysin/Rvs (BAR) domain proteins [13,14], which are recruited to the neck of the vesicle also help to stabilise membrane curvature and form a closed vesicle [15]. Release of the mature clathrin-coated vesicles is facilitated by the GTPase protein, dynamin, which is recruited to the neck of the budding vesicle via the BAR proteins [16]. After release from the plasma membrane, clathrin is dispersed from the vesicle by the ATPase activity of HSC70 [17] leaving the uncoated vesicle able to fuse to its target sorting endosome. One of the first examples of clathrin-mediated endocytosis was that of LDL, which binds to the LDLR clustered in clathrin-coated pits [18]. Interestingly, mutations in the cytoplasmic tail of the LDLR cause failure to cluster in clathrin-coated pits and are associated with familial hypercholesterolaemia (FH) [19].

### 2.2. Caveolae-Mediated Endocytosis

There are many forms of clathrin-independent endocytosis [4]. One such form involves caveolae (little caves) which assemble in cholesterol- and sphingolipid-rich nanodomains on the plasma membrane aided by the cholesterol-binding, caveolins [20]. Caveolins are integral membrane proteins found colocalised with many receptors [21] concentrated in caveolae. Binding of the ligand to its target receptor initiates endocytosis with the budding and release of the caveolae regulated by various kinases and phosphatases [22]. The dynamin protein also features in promoting membrane fission and release of the caveolin-coated vesicles [16]. Caveolin-mediated endocytosis is best known to be involved in transcytosis and mechano-sensing but also features in many cell signalling pathways, i.e., cell migration, cytokine, and beta-adrenergic signalling [23,24,25,26]. Caveolin-mediated signalling has been implied in the development of atherosclerosis [27] with the caveolin-1 protein displaying a role in the transcytosis of native LDL across vascular endothelial cells and the uptake of oxidised LDL (ox-LDL) in macrophages to form foam cells [27].

### 2.3. Macropinocytosis

Macropinocytosis is a form of pinocytosis that happens in all cell types enabling cells to imbibe extracellular fluid and nearby cargo in large endocytic vesicles known as macropinosomes [7,28]. The process of macropinocytosis is actin-driven requiring actin polymerisation beneath the plasma membrane to form membrane ruffles and a cup-like structure that closes at the margin, engulfing its cargo [7,29]. Macropinocytosis can be constitutive as is the case when cells utilise it for nutrient scavenging to promote growth or when immune cells sample their surroundings for antigens [28,30]. Constitutive macropinocytosis is dependent on the sensing of extracellular calcium by G-coupled receptors [28,30]. In several cell types, macropinocytosis is induced through the activation of growth factor, cytokine or Toll-like receptors which initiate the remodelling of membrane phospholipids [30,31]. Regardless of the initiating event, the mechanism by which macropinosomes form is thought to be similar, involving recruitment of Rac and Rho GTPases which drive the formation of actin networks and subsequent membrane extension and cupping [30]. Macropinocytosis has been shown to be involved in the uptake of ox-LDL by macrophages, thus leading to foam cell formation and subsequent development of atherosclerosis [29]. A recent paper posted on BioRxiv [32] has shown macropinocytosis to be involved in the uptake of lipoprotein(a) [Lp(a)], an LDL-like lipoprotein, in HepG2 cells which confers a similar risk to LDL in promoting cardiovascular disease [33].

### 2.4. Transcytosis

Transcytosis is the process by which macromolecules are transported across the cell from the apical to the bilateral surface (or vice versa) in polarised cells [34]. The initial phase of uptake is often via receptor-mediated endocytosis (either clathrin- or caveolae-mediated) [35], however, transcytosis can also take place after macropinocytotic uptake [5]. Regardless of the initial uptake process, the endocytosed molecule is subject to endosomal trafficking in transport vesicles via actin- or myosin-based motors through the intracellular space [5]. Along the way, other key proteins are recruited to the trafficking vesicle including the NSF/SNAP complex which engages SNARE proteins to trigger docking to the target plasma membrane and exocytosis of the transported molecule [5,35]. Many molecules have been shown to be transported via transcytosis, the most notable being albumin, insulin, and LDL [5]. The transcytosis of LDL particles across endothelial cells has been well documented and shown to be dependent on multiple LDL-binding receptors including the scavenger class B type 1 receptor (SR-B1) [36], and activin receptor-like kinase 1 (ALK1) [37]. A recent study utilising aortic endothelial cells and knockout mouse models [36] found LDL transcytosis to be mostly dependent on the SR-B1 receptor and showed that endothelial SR-B1 expression drives atherosclerosis in mice [36]. The authors also provided evidence that a guanine nucleotide exchange factor protein (DOCK4) regulated SR-B1′s internalization of LDL [36]. The SR-B1 receptor is more traditionally thought of as an HDL receptor which binds HDL and facilitates the uptake of lipids with HDL effectively outcompeting LDL for SR-B1 binding [38].

## 3. Plasma Lipoproteins

Lipoproteins are complex macromolecules of lipids and proteins present in plasma and the extravascular compartment [2]. They are composed of a hydrophobic core of lipids (triacylglycerols and cholesterol esters) surrounded by a single layer of hydrophilic phospholipids in which free cholesterol and apolipoproteins are embedded. Lipoproteins transport lipids between the intestine, liver and peripheral tissues [2]. Lipoproteins vary in their lipid and protein make-up, with the apolipoproteins being the prominent protein cargo either as an integral part of the assembled lipoprotein, i.e., apolipoprotein (apo)-B100, or acquired in circulation, i.e., apo-E. The apolipoproteins play a crucial role in lipoprotein metabolism with some acting as ligands for receptor-mediated endocytosis, i.e., apo-B100 and apo-E, and others acting as regulators of lipid-modifying enzymes, i.e., apo-C2 and apo-C3. Lipoproteins are classified according to their density into chylomicrons, very-low-density lipoproteins (VLDL), LDL and high-density lipoproteins (HDL), as they have been traditionally isolated using density ultracentrifugation [2].

### Pathways of Lipoprotein Endocytosis

**Figure 2:**
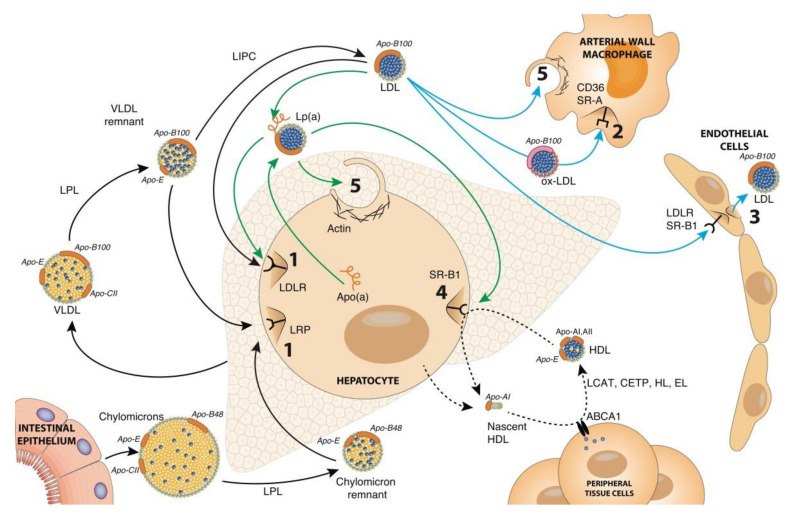
An overview of lipoprotein endocytosis pathways. Chylomicrons, assembled in the intestine from the absorption of dietary fat, are secreted into circulation where they are acted upon by lipoprotein lipase (LPL) activated by apo-CII to hydrolyse triacylglycerols. The released fatty acids are taken up by peripheral tissues and the resulting chylomicron remnant particles are taken up by hepatocytes through receptor-mediated endocytosis via an interaction with apo-E and the low-density lipoprotein receptor-related protein (LRP). Similarly, VLDL secreted by hepatocytes, also undergoes hydrolysis by LPL to form VLDL remnant particles, some of which are further hydrolysed by hepatic lipase (LIPC) to form LDL. The LDL particles are taken up by hepatocytes through receptor-mediated endocytosis via the low-density lipoprotein receptor (LDLR) binding to apo-B100. Native LDL can also undergo transcytosis in endothelial cells mediated by the SR-B1 receptor. Oxidised LDL (ox-LDL), which arises from oxidative modifications of native LDL, can be endocytosed by arterial wall macrophages either via CD36 or SR-A receptors. Macropinocytosis of native LDL particles by macrophages has also been shown to occur in a receptor-independent manner. LDL particles can form Lp(a) through disulphide bond formation between the apo-B100 and apolipoprotein(a) [apo(a)] components. Lp(a) uptake by hepatocytes can occur through the LDLR or SR-B1 receptor or through macropinocytosis. Nascent high-density lipoproteins (HDL) are secreted from hepatocytes in a lipid-poor state and interact with the ABCA1 transporter on peripheral cells to facilitate lipid transfer and formation of more mature HDL. The HDL molecules then undergo modification by various lipid-modifying enzymes (i.e., LCAT, CETP, HL and EL) and interact with the SR-B1 receptor to selectively transfer accumulated cholesterol esters back to the liver. Black arrows, major lipid transport pathways by apo-B-containing lipoproteins; green arrows, Lp(a) clearance pathways; cyan arrows, alternative LDL uptake pathways; dotted black arrows, HDL. (1) Receptor-mediated endocytosis, holoparticle uptake; (2) receptor-mediated endocytosis, modified holoparticle uptake; (3) transcytosis; (4) receptor-mediated endocytosis, lipid-only uptake; (5) macropinocytosis.

The metabolism of the apo-B-containing lipoproteins (Figure 2, black arrows) involves hydrolysis of core triacylglycerols by lipase enzymes anchored to cell surfaces followed by uptake of the hydrolysed particles by receptor-mediated endocytosis. Chylomicrons transport dietary fats, absorbed in the intestine, and VLDL transport endogenous lipids, synthesised in the liver. Both lipoproteins undergo a similar metabolic fate via lipoprotein lipase (LPL) on peripheral tissues hydrolysing their triacylglycerol load to release fatty acids for energy or lipid storage. The resulting chylomicron and VLDL remnant particles are taken up by clathrin-mediated endocytosis initiated via an interaction between apo-E and the low-density-lipoprotein-related receptor (LRP). A portion of the VLDL remnants is further metabolised by hepatic lipase (LIPC) on the liver surface to yield LDL which is taken up via clathrin-mediated endocytosis with apo-B100 acting as the ligand for the LDLR [39].

Many alternative uptake routes have been documented for LDL (Figure 2, cyan arrows). Native LDL has been shown to be transported by transcytosis, a process best documented in endothelial cells allowing LDL, with the aid of the SR-B1 receptor, to cross the arterial endothelial layer [36]. The LDL molecule has a propensity to be oxidised by various mechanisms to produce oxidised LDL (ox-LDL) [40] and in this form is subject to uptake by macrophages via the scavenger receptor-A (SR-A) and cluster of differentiation 36 receptor (CD36) receptors which bind to oxidised epitopes on apo-B100 [41,42]. Both SR-A and CD36 operate via clathrin-mediated endocytosis to promote oxLDL uptake with the endophilin-A2 protein increasing their expression and enhancing their oxLDL uptake activities [43]. Both native and ox-LDL have also been shown to be taken up by macropinocytosis, native LDL via a receptor-independent route and ox-LDL via a receptor-assisted route involving scavenger receptors [29].

Native LDL can also be metabolised in the form of Lp(a) (Figure 2, green arrows), a modified form of LDL assembled in circulation from the binding of LDL to the large apolipoprotein(a) [apo(a)] glycoprotein synthesised by the liver [44]. Multiple receptors have been shown to promote the uptake of Lp(a) including the LDLR and the SR-B1 receptors [45], although it is not clear if any constitute a major pathway for Lp(a) holoparticle uptake. A recent preprint [32] has demonstrated that macropinocytosis is an endocytic route for Lp(a) uptake with different receptors likely acting as anchors, rather than receptors, to initially bind the particle to the cell membrane.

The HDL molecule starts life as a lipid-poor particle secreted by the liver, which consists mainly of apo-A1 and phospholipid. Once in circulation, the particles interact with liver and peripheral cells (including macrophages) to acquire free cholesterol, a process mediated by the interaction of apo-A1 with the ATP-binding cassette A1 (ABCA1) cholesterol transporter [46,47]. The free cholesterol acquired via ABCA1 is esterified by the HDL resident enzyme, lecithin cholesterol acyltransferase (LCAT), to form mature HDL [47]. The lipid content of mature HDL is modified in circulation by cholesterol ester transfer protein (CETP), hepatic lipase (HL) and endothelial lipase (EL) [48,49]. The cholesterol esters in HDL are delivered back to the liver via selective lipid uptake by SR-B1 on hepatocytes [50]. The combined actions of SR-B1, HL and EL regenerate a lipid-poor particle for recirculation.

## 4. Approaches to Visualising Lipoprotein Endocytosis

Many different tools, from basic biochemical techniques to high resolution and live-cell imaging, have been used to study lipoprotein endocytosis to establish the molecules and compartments involved. The following provides a description of different approaches that have been applied from the early studies of Brown and Goldstein in their discovery of the LDLR pathway to recent in vivo imaging of lipoprotein uptake in whole animals. The LDL and Lp(a) molecules are used as examples and the advantages and disadvantages of the different tools used for visualisation are listed in Table 1. For a recent review on the pathways and approaches to studying HDL endocytosis we refer the reader to reference [2].

### 4.1. LDL Endocytosis

The observation that fibroblasts derive cholesterol specifically from LDL at low concentrations was the first clue that a specific receptor might be involved [57]. To confirm the presence of an LDL receptor, the protein component of LDL was labelled with ^125^I (^125^I-LDL) and incubated with normal and homozygous FH fibroblasts which fail to derive cholesterol from LDL [57]. These experiments, which were performed at 4 °C to prevent internalization, demonstrated normal cells to have high-affinity sites for LDL which were missing in the FH cells [57]. Subsequent studies showed the bound ^125^I-LDL was rapidly internalised within 10 min with the protein and cholesterol ester components degraded within 60 min [19]. The rapid degradation indicated lysosome involvement, which was confirmed with the lysosomal enzyme inhibitor, chloroquine [75]. The morphological features of the process were uncovered by electron microscopy studies using ferritin-coupled LDL [76] which revealed that the LDL was bound to areas in the plasma membrane that were coated and invaginated, suggesting endocytic vesicle formation. The coating was subsequently found to be clathrin [77]. Further, ^125^I-LDL binding assays using cycloheximide to inhibit LDLR synthesis revealed that the LDLR was likely recycled back to the membrane surface after internalisation [78]. This was later confirmed using monensin, an ionophore that blocks intracellular transport [79]. The LDLR was purified [80] and its partial sequence gained to allow for further biochemical and genetic studies of its structure and function [19]. It was during the dissection of the LDLR pathway that compactin, the precursor to the statin drugs now widely used to lower LDL levels, was identified [81,82].

The LDL pathway has been further dissected over the years to uncover many more regulators. One such regulator is protein convertase subtilisin/kexin type 9 (PCSK9), a secreted endoprotease which binds to the extracellular domain of LDLR and promotes its intracellular degradation [83]. Genetic studies of patients with mutations in PCSK9 had previously shown these patients to have altered levels of LDL [83]. Confocal fluorescence microscopy was used to visualise the effect of overnight incubations with PCSK9 on the uptake of LDL with a fluorescently labelled lipid component (Dil-LDL) in HEK293 cells [51]. Results showed a marked reduction in LDL uptake which was associated with a significant decline in LDLR protein levels (as determined by Western blotting) and a significant reduction in LDLR cell surface expression as identified by fluorescent cell sorting [51]. In the same study, confocal microscopy also found the Dil-LDL and fluorescently labelled PCSK9 proteins to be localised together in the lysosomal compartment in human hepatic carcinoma (HepG2) cells [51]. These results indicated a major role for PCSK9 in degrading the LDLR. An understanding of the regulatory role of PCSK9 on LDL endocytosis has led to the development of PCSK9 inhibitors which are now in clinical use for lowering LDL [84].

Further players recently identified in regulating the endocytosis of LDL are the protein complexes COMMD/CCD22/CCDC93 (CCC) and Wiskott-Aldrich syndrome protein, and SCAR homologue (WASH) [85]. The CCC complex is known to interact with WASH to regulate the recycling of membrane transporters [86]. The WASH protein complex has been shown to drive the actin filament formation required for endosomal sorting [87]. Both complexes interact with, and are recruited by, the retromer complex which mediates protein trafficking and sorting [86]. Deficiencies in COMMD1 have been associated with elevated LDL cholesterol levels in both human and animal models [70] and a mutation in one of the protein components of WASH was found to be associated with elevated LDL cholesterol in affected individuals [70]. Immunoprecipitation experiments in HEK293 cells revealed an interaction between COMMD1 and the LDLR which was further confirmed in mouse embryonic fibroblasts (MEFs) where the COMMD1 and LDLR proteins were found to be colocalised via confocal fluorescence microscopy [70]. Similar results were obtained with the WASH1 and LDLR proteins [70]. Depletion of either COMMD1 or WASH1 caused a significant reduction in LDLR surface localisation [70] as determined by surface biotinylation assays. Results indicated that a deficiency in either protein complex caused the LDLR to be missorted to the lysosome for degradation instead of being recycled [70,85]. Knockout of either protein in MEFs was associated with a significant impairment in Dil-LDL uptake as visualised by confocal fluorescence microscopy [70] confirming both proteins to be involved in LDL endocytosis.

As well as the classic receptor-mediated pathway, LDL has been reported to be endocytosed by transcytosis [52]. Armstrong and co-workers employed a novel assay based on total internal reflection fluorescence (TIRF) microscopy [52] to visualise transcytosis of Dil-LDL across primary human coronary artery endothelial cells (HCAECs) showing the process to be inhibited by both unlabelled LDL and a dynamin inhibitor. They then demonstrated a role for SR-B1 in LDL transcytosis by overexpressing SR-B1 which significantly increased Dil-LDL transcytosis and transfecting with a siRNA to SR-B1 which had the opposite effect. Furthermore, using spinning disk confocal microscopy they showed that SR-B1 partially colocalised with Dil-LDL [52]. The importance of SR-B1 for transcytosis of LDL was further confirmed in an extensive study utilising an endothelial-specific SR-B1 knockout mouse model [36]. A reduction in incorporation of intravenously administered Dil-LDL was evident in SR-B1 knockout aortas when compared to the wild type, as identified by confocal fluorescence microscopy. This was corroborated by a significantly reduced apo-B signal in aortic homogenates as detected by Western blotting in the knockout compared to wild-type mice. Furthermore, macrophages from the SR-B1 knockout arteries isolated by flow cytometry demonstrated significantly less Dil-LDL fluorescence than the wild type.

Kruth and colleagues have demonstrated that native LDL is taken up by macrophages to form foam cells in a receptor-independent manner [88]. Using time-lapse phase-contrast video microscopy, they demonstrated that PMA-stimulated human monocyte-derived macrophages internalise LDL by macropinocytosis [88] and that the uptake was not prevented by inhibition of receptor-mediated uptake. In a subsequent study using M-CSF-activated murine bone marrow-derived macrophages [89], similar findings were presented using a range of macropinocytotic inhibitors and independence from the LDLR, SR-A and CD36 receptors was demonstrated confirming receptor-independent macropinocytosis as another uptake pathway for LDL.

### 4.2. Lp(a) Endocytosis

Lp(a) is assembled in circulation from LDL binding to the highly glycosylated plasminogen homologue, apo(a) produced in the liver [44]. The main site for Lp(a) catabolism is the liver [58] and its similarity to LDL set the expectation that its clearance from circulation would be via the LDLR. Havekes and co-authors [90] provided convincing evidence of this when they demonstrated similar binding curves between ^125^I-LDL and ^125^I-Lp(a) to fibroblasts and competition between both lipoproteins for binding sites. They went on to show by electron microscopy that ^125^I-Lp(a) was bound in coated pits on the cell membrane. Subsequent studies utilising Western blots to detect Lp(a) internalisation have suggested catabolism of Lp(a) by the LDLR and have shown its dependence on clathrin and PCSK9 [91,92]. However, mouse studies have provided conflicting evidence with one study documenting that mice overexpressing the LDLR removed Lp(a) from the plasma at a greater rate than wild-type mice [53] and another study reporting LDLR knockout mice to have a similar clearance of ^125^I-Lp(a) to wild type [58]. Similar conflicts occur in human studies in FH subjects lacking a functional LDLR, with some studies documenting higher levels of Lp(a) [93] and others reporting no significant difference in Lp(a) levels between FH and non-FH subjects [94].

A role for SR-B1 in Lp(a) endocytosis was documented by Yang et. al. [66] where, using flow cytometry, they showed an increase in the association of Alexa Fluor 488-labelled Lp(a) to HEK293 cells stably transfected with SR-B1 compared to untransfected controls. Confocal fluorescence microscopy of HeLa cells transfected with SR-B1 demonstrated cell-bound Alexa Fluor 488 Lp(a) and both apo-B and apo(a) internalisation which was not apparent in control cells. Labelling of the Lp(a) cholesterol component with BODIPY-CE also showed a selective uptake of Lp(a) cholesterol which was in line with SR-B1′s known function of selective lipid uptake. Transgenic mouse studies confirmed a role for the SR-B1 receptor with overexpression of the receptor enhancing Lp(a) uptake, and knockout of the receptor reducing uptake [66]. Interestingly, another study documented the selective uptake of phospholipids from Lp(a) by SR-B1 in HepG2 cells after antibody and chemical blocking of SR-B1 [95]. There is also some evidence that another scavenger receptor, CD36, and its partner, Toll-like receptor 2 (TLR2), may be involved in mediating the uptake of oxidised phospholipids from Lp(a) in macrophages [66].

Lp(a) endocytosis has also been reported to involve plasminogen receptor (KT) (PlgRKT) [54]. Western blotting showed increased internalisation of Lp(a) in HepG2 and HAP1 cells overexpressing PlgRKT and, contrariwise, a reduction in Lp(a) uptake in HAP1 PlgRKT knockout cells. Confocal fluorescence microscopy showed colocalisation of endogenous PlgRKT and Lp(a) in Lp(a)-treated HepG2 cells [54]. Treating the cells with Dil-Lp(a) and subsequently tracking the Dil-LDL component and the apo(a) component (detected by immunostaining) revealed the two to diverge after internalisation, with the LDL being trafficked to the lysosome [54]. Immunostaining of apo(a) and various endosomal markers revealed that the apo(a) component was trafficked via early endosomes and the trans-Golgi network to recycling endosomes which was corroborated by detecting recycled apo(a) in the media [54]. The recycling of apo(a) was also apparent in human dermal fibroblasts cells using similar methods [54]. These studies demonstrated a role for PlgRKT in Lp(a) uptake and uncovered a novel recycling pathway for apo(a).

While the involvement of the LDLR and SR-B1 receptors align with receptor-mediated forms of Lp(a) uptake, in the case of PlgRKT it is not certain whether it acts as a receptor or whether it acts as an anchor to bind Lp(a) to the cell surface ahead of endocytosis. A recent study suggests that Lp(a) is internalised by macropinocytosis [32]. Using various inhibitors of macropinocytosis, the uptake of Lp(a) was significantly reduced, as quantified by confocal imaging of immunostained Lp(a) vesicles. Interestingly, a recombinant apo(a)-mScarlet was shown to respond similarly to Lp(a) suggesting that the apo(a) moiety was mediating macropinocytosis. From studies to date it is becoming clear that Lp(a), like LDL, is subject to many different forms of endocytosis.

## 5. Live Imaging of Lipoprotein Endocytosis

Live cell imaging of lipoprotein endocytosis can be achieved with the use of non-toxic stable dyes incorporated into the lipoproteins or by DNA technologies to fluorescently tag proteins of interest within the lipoproteins and cellular compartments. Both labelling methods obviate the need to use antibodies and avoid the destructive methods that come with antibody detection. Combined with the use of an incubation chamber mounted to a high-resolution microscope, such as a confocal or spinning disk, lipoprotein uptake can be monitored while gaining temporal, spatial and morphological information. There are very few reports of live-cell imaging of lipoprotein endocytosis and trafficking. One report utilised a green fluorescent protein (GFP)-tagged apo-E construct to visualise the trafficking of apo-E-containing vesicles via confocal microscopy during secretion from human monocyte-derived macrophages [96]. Another report utilised an apo-E-GFP fusion protein to monitor lipoprotein egress from Huh-7.5 liver cells [97]. After confirming colocalisation of the apo-E-GFP with apo-B by confocal microscopy to indicate incorporation into lipoproteins, the trafficking of apo-E-GFP was visualised in real-time on live single cells using TIRF microscopy [97]. A recent study utilised pHrodo red-labelled LDL to visualise LDL internalisation over time in different human cell lines [74]. Human renal epithelial (HK2), HepG2 and HCAEC cells were monitored over 4 h by fluorescence microscopy with serial measurements to visualise the uptake of LDL over time [74]. The authors validated the system by showing that LDL internalisation was sensitive to well-known inhibitors of LDL uptake, i.e., dynasore and recombinant PCSK9, as well as promoters of uptake, i.e., simvastatin. While the type of microscopy used in this study was of low resolution and unable to provide localisation information, the protocol allows for a high throughput screening for modulators of lipoprotein uptake while also assessing cell toxicity [74]. Coupled to a higher resolution microscope, it could be utilised to gain localisation information and establish the role of various proteins throughout different stages of lipoprotein endocytosis.

An exciting new study has described the visualisation of lipoproteins in whole animals. Thierer et. al. [98] reported the imaging of lipoproteins in zebrafish after targeting an engineered luciferase reporter (NanoLuc) into the endogenous apo-B gene locus, essentially tagging all apo-B-containing lipoproteins as fluorescent. Several genetic, pharmacological and dietary manipulations known to affect lipoprotein metabolism were applied to live zebrafish to prove that reproduced “LipoGlo” lipoprotein particles responded to the treatments. The LipoGlo signal and localisation patterns throughout these treatments were visualised using LipoGlo chemiluminescent microscopy on whole-mount images of zebrafish larvae. The authors concluded that the new LipoGlo system showed the lipoprotein profiles of zebrafish to be human-like in their responses to various treatments and that the model could prove useful for the high-throughput live-animal screening of modulators of atherogenic lipoproteins.

## 6. Conclusions

Endocytosis comes in many forms providing cells with precious cargo for processing and supplying the cell with nutrients and cues for driving downstream cellular actions. One class of molecules that have been well-studied with respect to their uptake by endocytosis is the plasma lipoproteins which are internalised using many different forms of endocytosis. Several approaches have been applied to visualise lipoprotein internalisation, from basic biochemical approaches to in vivo imaging, with high-resolution live-cell imaging holding much promise for the future. Much has been gained from these studies in dissecting the molecules involved. In the case of LDL, a thorough understanding of the LDLR-mediated endocytosis pathway has provided crucial information for the characterisation and diagnosis of patients with gene defects in the LDLR and other regulators of the pathway. It has also established a well-trodden platform for the development of drugs targeting the LDLR to lower LDL and reduce the risk of developing atherosclerosis, i.e., statins and the PCSK9 inhibitors. The more complex Lp(a) molecule, like LDL, appears to also undergo many forms of endocytosis, although more studies are needed to establish if any of these pathways could be targeted for therapies to lower Lp(a). Studies of the endocytic routes responsible for the uptake of the LDL and Lp(a) molecules into arterial cells, including resident macrophages, have also been fruitful in providing information on pathways promoting atherosclerosis. No doubt new players regulating the many routes of lipoprotein endocytosis will be identified and new drug targets pinpointed as the pathways are visualised to finer detail in the future.

## Figures and Tables

**Table 1:** Approaches for visualising lipoprotein endocytosis.

Technique	Advantages	Disadvantages	References
Western Blotting	Accessible to all researchers Specificity Sensitivity	Specificity and sensitivity are antibody dependent Low throughput Time inefficient No information of location and morphology	[51,52,53,54,55,56]
^125^I binding assays	High sensitivity Allows to track binding and degradation of ligands	Limited information of location and morphology Requires preparation of labelled lipoproteins	[38,55,57,58,59,60,61]
Electron microscopy	High resolution High range of magnification Greater depth of field Visualises cell architectures and small structures	High cost of equipment Hard to distinguish between different structures Used only for fixed or frozen materials More prone to imaging artefacts Elaborate sample preparation	[59,62,63,64,65].
Flow cytometry	High throughout Specificity Sensitivity	Limited information of location and morphology Specificity and sensitivity are antibody dependent	[66,67,68,69]
Confocal microscopy	Adjustable depth and a capability for serial section imaging Multicolour lasers allow experiment coupling Determination of molecular localisation Assessment of cellular morphology	Limited number of excitation wavelengths High cost of purchasing and operating the system Prone to bleaching and photodamage during prolonged sessions or high laser power	[51,54,55,66,67,68,70,71,72]
Total internal reflection fluorescence (TIRF)	Increased duration of experiments Reduced phototoxic stress and photobleaching	Only adherent cells can be used	[52,56,73]
Live cell microscopy	Less prone to imaging artefacts Couples temporal, spatial, and morphological information	Accurate environmental conditions need to be maintained to preserve cellular activity Prone to bleaching and photodamage during prolonged sessions or high laser power	[74]

## Data Availability

Not applicable.
